# Evaluation of mandibular trabecular and cortical bone by fractal analysis and radiomorphometric indices in bruxist and non-bruxist patients

**DOI:** 10.1186/s12903-023-03245-y

**Published:** 2023-07-25

**Authors:** Mesude Çitir, Hazal Karslioglu, Canan Uzun

**Affiliations:** 1 Department of Dentomaxillofacial Radiology, Faculty of Dentistry, Tokat Gaziosmanpasa University, Tokat, Turkey; 2 Department of Dentomaxillofacial Radiology, Faculty of Dentistry, University of Baskent, Ankara, Turkey; 3 Department of Dentomaxillofacial Radiology, Faculty of Dentistry, University of Health Sciences, İstanbul, Turkey

**Keywords:** Bruxism, Fractals, Index, Mandible, Panoramic, Radiography

## Abstract

**Background:**

The aim of this study was to evaluate the effect of bruxism on the cortical and trabecular bone of the mandible using the radiomorphometric indexes and fractal analysis (FA) additionally to examine the efficiency of FA as diagnostic test for bruxism.

**Methods:**

Evaluation was performed on panoramic radiographs of 94 bruxists and 94 non-bruxist individuals with the ImageJ program. Cortical bone was assessed with mandibular cortical index, mental index, and panoramic mental index. Trabecular bone in the condyle, gonial, and corpus region was evaluated by FA. An independent sample t and Mann-Whitney-U tests and Pearson and Spearman rank correlations were conducted for statistical analysis.

**Results:**

A total of 188 participants, 112 female, and 76 male, were included in the study. The sample age ranged from 18 to 43, with a mean of 27.55 (± 7.022) years. FA values of the angulus were significantly higher than those of the condyle and corpus, and the mean of the sample for the angulus, condyle, and corpus, respectively, were; 1.36 (± 10), 1.10 (± 0.9), 1.13 (± 0.8). There was a positive correlation between FA of the mandibular corpus and age (*r* = .163, *p* = .025). Females’ values were smaller than males’ in the FAs of three regions, and significant differences were found in FA of the condyle and angulus of the mandible, MCI, and PMI according to gender. There was no statistically significant difference between bruxist and non-bruxist patients in term of FAs of three regions, MCI, MI, and PMI values (*p* > .05).

**Conclusions:**

FA of the condyle and angulus of the mandible, MCI, and PMI are significantly affected by gender. However bruxism doesn’t cause a significant change in the fractal dimensions of the bone in the mandible and doesn’t change substantially MCI, MI, and PMI.

## Background

Bruxism is a repetitive jaw-muscle activity characterized by clenching or grinding of the teeth and/or by bracing or thrusting of the mandible during sleep or wakefulness [1]. Although it has been associated with various psychosocial, physiological, and exogenous factors, there is no consensus on the etiology [2]. Bruxism can be detrimental to the temporomandibular joint, and cause myofascial pain, and may be responsible for some complications in dental implants and implant-supported prostheses [2–4]. Such sustained continuous and excessive forces also have some effects on the bone, which may be visible radiographically [5]. Continuous forces transmitted from muscles to the bone cause change in bone mineral density [6, 7] and guide bone remodeling through resorption and apposition activities [8, 9]. This raises the possibility that bruxism causes morphological changes in jaws. Panoramic radiography (PR), which reveals the jaws bilaterally, is the first and most commonly used imaging technique when examining the jaws. Although PR has disadvantages due to its being a 2-dimensional imaging method, it is indispensable in dentistry with advantages such as requiring a lower radiation dose, easy access, and interpretation compared to 3-dimensional imaging systems [10]. PR can give an idea of the health of the cortical bone of the mandible [11, 12] and allows the assessment of vertical dimensions in the mandible [13, 14].

Fractal analysis (FA) is a method that has been studied in many studies in image analysis problems to detect early information about the bone mineral status [15]. PR is the most commonly used imaging modality when examining the FA of the jaws [16]. FA values on PR of osteoporotic patients have been studied several times, and a recently published meta-analysis reported that FA analysis of the mandible on PR had a sensitivity of 86.2% and a specificity of 72.7% in osteoporosis screening [17]. Determining the cut-out fractal dimension (FD) of certain regions in mandible corresponding to the onset of degeneration would be an important parameter in the diagnosis of some diseases. Although we do not have this information yet, the data to be added to the literature on this subject will contribute to the knowledge of the normal FD range of the jaws.

One aim of this study was to investigate the effects of bruxism on the morphology and bone density of the mandible by the mandibular cortical index (MCI), mental index (MI), panoramic mandibular index (PMI), and FA. To understand whether these indexes and FA values differ significantly in bruxist individuals compared to the control group. Another purpose of the study was to examine the efficiency of MCI, MI, and PMI with FA as a diagnostic test for bruxism. This is the first study to evaluate these two disciplines in the assessment of bruxism. This will provide an opinion on whether FA is more sensitive than indices in showing changes in the bone in bruxist patients. The null hypothesis was that FA values of the condylar, corpus, and gonial region and MCI, MI, and PMI were not different in bruxist and non-bruxist subjects.

## Methods

### Study design and patient selection

This prospective study contains individuals who applied to the dentomaxillofacial radiology clinic of the dentistry faculty hospital and volunteered to participate. The study was approved by Tokat Gaziosmanpaşa University Faculty of Medicine Clinical Research Ethics Committee (21-KAEK-015) and complied with the principles of the Declaration of Helsinki. Only systemically healthy individuals who had angle class I occlusion with natural teeth and no missing teeth (except third molars) were included in the study. Informed consent was obtained from all patients to be included in the study. According to anamnesis and clinical examination, two groups were formed as bruxist and non-bruxist. Patients with systemic or metabolic disease and with a cyst, tumor, dysplasia, or any other bone lesion in their jaws were excluded from the study and control groups. Neurological and psychiatric disease, drug or alcohol use, and previous or ongoing orthodontic treatment were also reasons for exclusion. The individual was not included in the study when the presence of deep caries, moderate to severe periodontal bone loss, or any other pain foci, which could lead to a change in occlusion, in the tooth and periodontium, was determined. Additionally, all individuals included in the study had no joint problems and had no joint related pain, joint sound or dysfunction in temporomandibular joint.

The power analysis indicated 188 individuals for the sample size, for a power of 87.7%. Accordingly, the study group as bruxists and the control group without evidence of bruxism were formed from 94 individuals each. The distribution of males and females was planned equally within these groups, and the age range was restricted between 18 and 45.

### Clinical examination

The diagnosis of bruxism was made by evaluating the information obtained from the anamnesis and the findings obtained during the clinical examination. Attrition on functional and non-functional tubercles, linea alba prominence and teeth marks on the tongue edge, and the pain and tenderness in the masticatory muscles were examined clinically. In the anamnesis, it was questioned if the individuals clenched or grinded their teeth during the day or night, whether the sleep partners reported tooth grinding noise, and if any pain or fatigue was present in their jaws when they woke up or during the day. The presence of at least one of the anamnesis information, along with tooth wear and/or tenderness in the chewing muscles during the clinical examination was considered as bruxism. The same author with seven years of experience examined all the patients in the study and took their anamnesis (MC).

### Imaging and display features

All PRs were obtained using a Veraviewepocs 2-D (J. Morita Mfg. Corp., Kyoto, Japan) digital panoramic x-ray unit, operated automatically 67 kVp according to patient size, 5 mA, and exposure time of 7.4 s. PRs that failed to display adequate diagnostic image quality due to artifacts or positioning errors, were not included in the study. Radiographs were evaluated under dim light and on 27-inch Dell Precision T3620 medical monitors (Dell, Round Rock, TX, USA) with 1920 × 1200 pixel resolution and 64-bit color support by one of the authors (HK). The dentomaxillofacial radiologist repeated the assessments after a 3-weeks interval to quantify intraobserver agreement. Both linear measurements and FA were performed on images, transferred in JPEG (Joint Photographic Experts Group) format, using ImageJ v 1.53-win-java8 software, a version of NIH Image (US National Institutes of Health, https://imagej.nih.gov/ij/download.html) with 150% magnification.

### Radiographic measurements

**Fig. 1:**
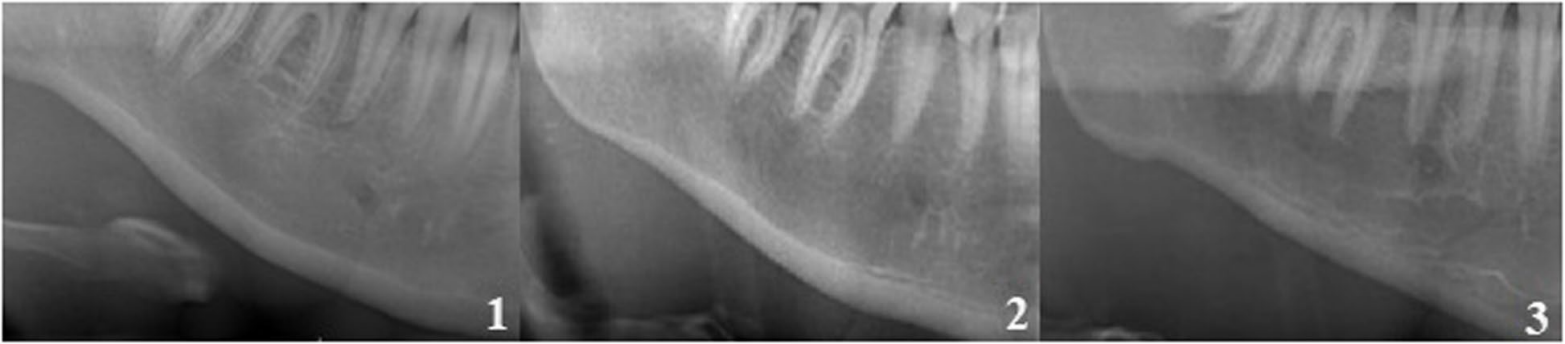
Representation of the Mandibular Cortical Index (MCI) according to Klemetti (1994). 1) normal cortex appearance with even and sharp endosteal margins on both sides, (2) semilunar defects and/or endosteal cortical residues on one or both sides of the endosteal margin of the cortex 3) severe endosteal cortical residues and porosity in the cortical layer

MCI was used to evaluate bone density. In the MCI, inferior cortex of the mandible was divided into three categories: (1) normal cortex appearance with even and sharp endosteal margins on both sides, (2) semilunar defects (lacunar resorption) and/or endosteal cortical residues are present on one or both sides of the endosteal margin of the cortex (3) severe endosteal cortical residues and porosity in the cortical layer (Fig. 1) [18].

**Fig. 2:**
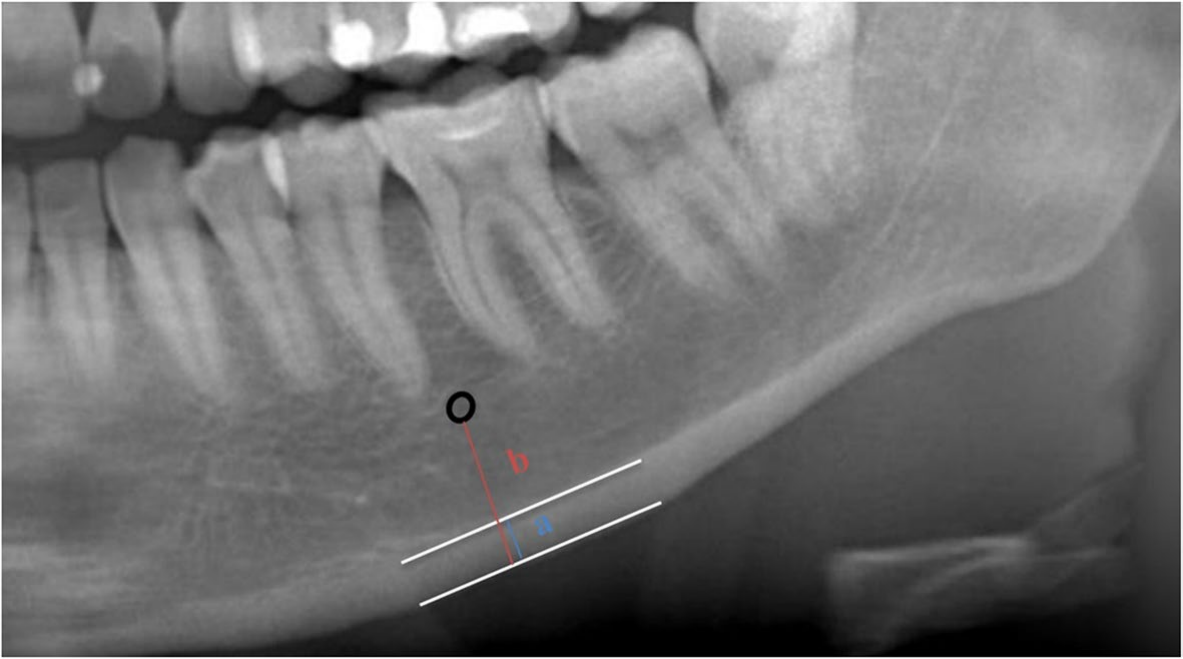
Measurement of the mental index (MI) and panoramic mandibular index (PMI). MI; the cortical width at the mental foramen region (**a**), (PMI); the ratio of the mandibular cortical width to the distance between the lower border of the mental foramen and the inferior border of the mandible (**a**/**b**) (Ledgerton 1997)

Quantitative assessment of the mandible was made by MI and PMI. In the MI, cortical width was measured at the line of the mental foramen. Cortical width is measured in a perpendicular line from the mental foramen to the inferior border of the mandible (Fig. 2a) [19]. In the PMI, the ratio of the mandibular cortical width to the distance between the lower border of the mental foramen and the inferior border of the mandible was taken (Fig. 2a/b) [20].

### Fractal analysis

**Fig. 3:**
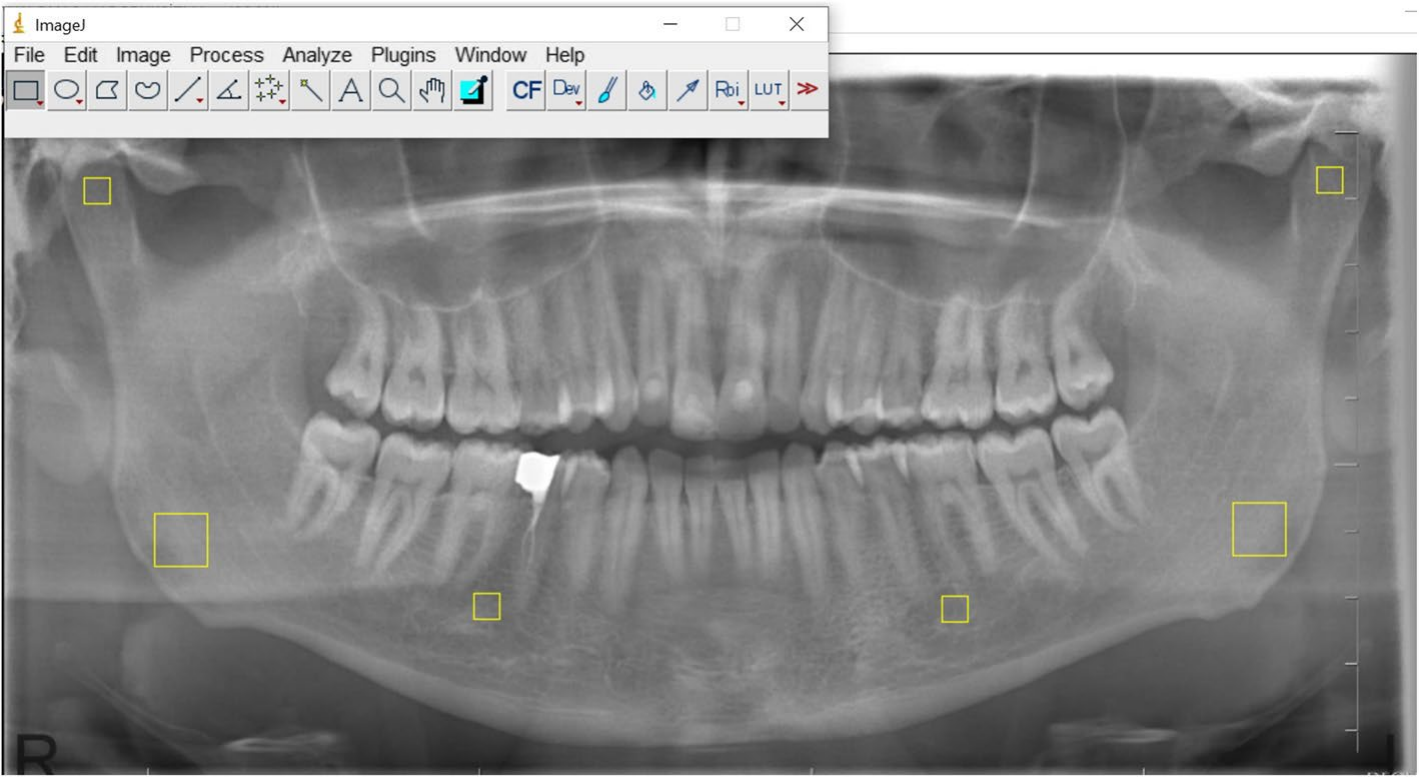
Selection of regions of interest (ROIs) in the condylar region, in the mandibular corpus and the gonial region

FA was performed bilaterally on three different regions of interest (ROIs) as follows: (1) 25 × 25 pixel in the condylar region, (2) 25 × 25 pixel in the mandibular corpus between the second premolars and the first molars (3) 50 × 50 pixel in the gonial region (angulus mandible)(Fig. 3).

The periodontium of teeth and the cortical border of the mandible and the mandibular canal were excluded in all ROIs.

**Fig. 4:**
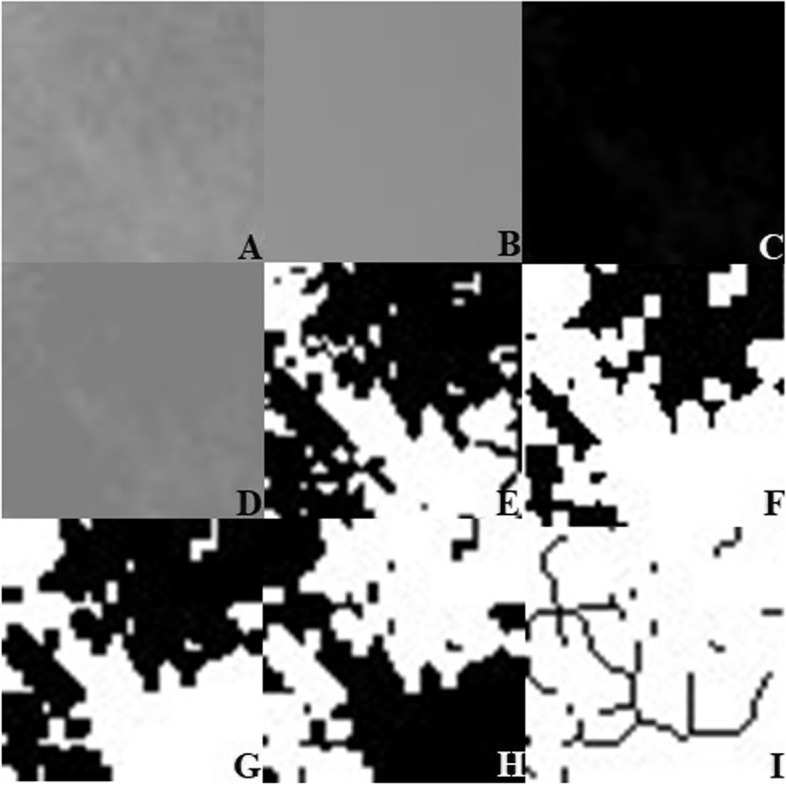
Fractal analysis steps; **a**) Duplication of ROI **b**) Blurr with Gaussian filter **c**) Subtraction **d**) Addition of 128 grey value **e**) Binarization **f**) Erosion **g**) Dilatation **h**) lnvertion **i**) Skeletonization

FA was performed according to the box counting method defined by White and Rudolph [21] as follows: The selected regions from the PR were cropped and duplicated. Then the duplicated image was blurred with Gaussian filter (sigma:35 pixel) to get rid of the brightness variations due to overlying soft tissues and varying bone thicknesses. The blurred image was subtracted from the original image, and a grayscale value of 128 was added to distinguish between bone marrow spaces and trabeculae. The image was converted to 2-color format as black and white using the “Threshold” option. The noise of the resulting image was eliminated with the “Erode” option and the outer lines of the structures were made more visible with the “Dilate” option. With the “lnvert” option on the image, the outline of the trabecular bone was revealed by turning the white areas into black and the black areas into white. The outlines of the trabecular structure were determined skeletally by lines for FA with the “Skeletonize” option. Then, using the “Fractal box counter” option, FD values were calculated from the slope of the line filtered at the data points. At this stage, the image is divided into squares of 2, 3, 4, 6, 8, 12, 16, 32 and 64 pixel dimensions. The number of squares containing the trabeculae and the total number of squares were calculated for each pixel. These values were displayed on a logarithmic scale, and the slope of the line gave the FD value (Fig. 4).

### Statistical analysis

SPSS software version 25.0 (IBM Corp., Armonk, NY, USA) was used to analyze the data. Data were analyzed by descriptively using mean and standard deviation for numeric data and frequency and a percentage ratio for categorical data. The normality of the continuous numerical variables was analyzed by skewness and kurtosis, followed by an independent sample t-test or Pearson correlation coefficient to evaluate the data normally distributed between the two variables, and Mann-Whitney-U test or Spearman rank correlation coefficient were applied to evaluate the data non-normally distributed. Intraobserver reliability was assessed by using Pearson correlation (for MI, PMI, and FA results) and Cohen’s kappa coefficient (for MCI). Statistical significance was set at the *P* < .05 level in all analyses.

## Results

The first and second measurements of the observer were highly correlated at 0.000 significance level for FA of condyle, angulus, and corpus, MI, and PMI in each side (r values were between 0.899 and 0.999). Intraobserver agreement of both right and left MIC was found to be almost perfect (k values were 0.86 and 0.81 respectively, *p* < .000) [22].

A total of 188 individuals, 94 of whom were bruxists and 94 non-bruxists, were included in the study. The sample age ranged from 18 to 43, with a mean of 27.55 (± 7.022) years. The mean age was 27.48 (± 7.651) in the bruxist group and 27.63 (± 6.407) in the non-bruxist group. Age distribution was statistically equivalent in the bruxist and non-bruxist groups, (t(180.67)=-0.145, *p* = .885).

Of the 188 participants in the present study, 112 were female, and 76 were male. The gender distribution was the same in the bruxist and non-bruxist groups, with 56 (59.6%) females and 38(40.4%) males.

A significant correlation was observed between FA values of the right and left condyle, angulus, and corpus regions of all participants and the MCI, MI, and PMI of the right and left sides (Table 1). Then, the mean values of the right and left sides were taken and continued with a single value per person.

**Table 1:** Correlation between FA values, MCI, MI, and PMI of the right and left sides

	r	p
Condyle	.177	.015
Angulus	.455	.000
Corpus	.195	.007
MCI	.667	.000
MI	.776	.000
PMI	.594	.000

Among the variables examined, only FA of the mandibular corpus was significantly but weakly correlated with age in all participants (*r* = .163, *p*=(0.025). That is, an increase in FA of the mandibular corpus was determined with the increase in age.

When we evaluated the variables according to gender, significant differences were found in FA of the condyle and angulus of the mandible and also MCI and PMI (Tables 2 and 3). The difference in FA of the mandibular corpus and MI remained below statistical significance. Females’ values were smaller than males in the FAs of three regions and in indices other than PMI, while PMI values were smaller for males.

**Table 2:** Comparison of the mean values of FA and MCI in the mandible according to gender

	Gender	Mean with std. deviation	T	P
FA-condyle	female	1.08 ± 0.08	-2.707	.008*
	male	1.12 ± 0.08		
FA-angulus	female	1.34 ± 0.10	-2.452	.012*
	male	1.38 ± 0.09		
FA-corpus	female	1.12 ± 0.08	-1.290	.199
	male	1.14 ± 0.08		
MCI	female	1.50 ± 0.49	-4.530	.000*
	male	1.87 ± 0.62		

*p < .05 is significant

**Table 3:** Comparison of the median values of MI and PMI in the mandible according to gender

	Gender	Median (min & max)	U	P
MI	female	3.61 (1.82 & 6.53)	3863.50	.284
	male	3.74 (1.02 & 5.83)		
PMI	female	.59 (0.25 & 1.98)	3211.50	.004*
	male	.52 (0.23 & 1.05)		

*p < .05 is significant

FA values of the condyle, angulus, and corpus regions did not correlate with each other. (Both in the mean values of the right and left side and the values from same side of the jaw, *p* > .05). FA values of the angulus were higher than those of the condyle and corpus, and the mean of the sample for the angulus, condyle, and corpus respectively were: 1.36 (± 0.10), 1.10 (± 0.09), 1.13 (± 0.08).

When we evaluated the FA values according to bruxism, both in general sample and among females and males, the difference in mean of FA values for all three regions remained below statistical significance (*p* > .05). When we analyzed the indices according to bruxism, it was seen that the differences in MCI, MI, and PMI of bruxist and non-bruxist groups, both in the general sample and among females and males were below statistical significance (*p* > .05). Thus, the null hypothesis was not disproved. Bruxism analysis results are available in Tables 4 and 5.

**Table 4:** Comparison of the mean values of FA and MCI in the mandible according to bruxism

	Bruxism	Mean with std. deviation	T	P
FA-condyle	present	1.11 ± 0.09	1.279	.202
	absent	1.09 ± 0.08		
FA-angulus	present	1.35 ± 0.10	-1.522	.130
	absent	1.37 ± 0.10		
MCI	present	1.63 ± 0.54	-.504	.615
	absent	1.67 ± 0.60		

*p* < .05 is significant

**Table 5:** Comparison of the median values of FA, MI and PMI in the mandible according to bruxism

	Bruxism	Median (min & max)	U	P
FA-corpus	present	1.13(0.92 & 1.30)	4369	0.895
	absent	1.15 (0.84 & 1.29)		
MI	present	3.48 (1.56 & 6.53)	3771	0.083
	absent	3.77 (1.02 & 6.47)		
PMI	present	0.54 (0.23 & 1.25)	4042	0.313
	absent	0.58 (0.29 & 1.98)		

*p* < .05 is significant

## Discussion

In this study, there was no statistically significant difference between bruxist and non-bruxist patients in term of FA values and index analyses. Although a significant correlation was found in the FA values of ROIs selected from the same region on individuals’ right and left sides, no such agreement was found in the FAs of ROIs selected from different anatomical regions on the same side.

In this study, FA values of the angulus were higher than those of the condyle and corpus. Gulec et al. [23] also found the highest mean FA values for the gonial region in their study including gonial, condylar, and dentate regions. The angulus region is where the masseter muscle attaches to the mandible and is subject to high muscle activity. Lee et al. [24] reported that decreased muscle activity with botulinum toxin type A injection into the masseter muscle in patients with masseteric hypertrophy affects the mandibular angle as a decrease in bone volume. A positive relationship between muscle activity and muscle mass and bone mineral density has also been reported [25, 26], and the higher FD values have been associated with an increase in the complexity of the structure and more trabeculation in the literature [21, 27].

Eninanç et al. [28] reported significantly lower FA for the gonial region in bruxist individuals than in control individuals, however, there was no significant difference between the two groups in the condylar and dentate regions. This finding has been attributed to the degenerative effect of repetitive contraction forces in bruxism on the gonial region because it has already been reported that decreased FA measurements are closely associated with increased bone demineralization [27]. However, the reaction of the bone in bruxism seems different. A recently published study reported that individuals with bruxists were 300 times more likely to have bone apposition in the gonial region than non-bruxists [29], and two another study reported that an association between the presence of bony exostoses at the mandibular angle and bruxism [30, 31]. Additionally, in two studies involving pediatric patients and adults, significantly higher FA values for the angulus were reported in bruxist patients than in non-bruxist patients [32, 33].

Arsan et al. [34] reported that mandibular condyles with temporomandibular disorders had significantly lower FA values than those of healthy temporomandibular joints, and FA values decreased as the severity of degenerative changes increased on the left side. On the other hand, they found a significant difference between FAs of condyles with gross erosive changes and those with mild erosive changes, but not between condyles with gross erosive changes and condyles with normal appearance [34]. Another study also reported significantly lower FA measurements for the right condyle in bruxist individuals than in non-bruxist individuals and no significant difference for the left condyle [23]. Kolcakoglu et al. [32], in contrast, reported significantly higher FA values in the condyle of bruxist pediatric patients than in non-bruxist children. A high prevalence of degenerative changes in the mandibular condyles has been reported in individuals with bruxism [35]. In the condyle, when the bone is exposed to increasing forces, it may become osteoproliferative, as in osteosclerosis and osteophyte formation or there may be a decrease in bone mass, as in erosion and subchondral cyst. There is a study reported a detectable decrease in trabecular bone density in the condyle of females who underwent botulinum toxin A injections for their masticatory muscle pain [36]. On the other hand, Arsan et al. [34] reported the lowest FA values for both erosive and sclerotic condyles among the degenerative changes, one of which (erosion) was more common in their study in patients with temporomandibular disorders and the other in healthy controls. Although these are opposite reactions of the bone, a similar differentiation of trabecular architecture may be possible for both the erosive and the sclerotic condyle, such as decrease in complexity and trabeculation and transformation to more simple form.

Gulec et al. [23], reported statistically lower FA values in the right and left gonion and left condyle in females and no difference according to gender in the right condyle. In the study of Eninaç et al. [28], FA values in right and left condylar regions were lower in females than in males in both bruxist and non-bruxist individuals and FA values in dentate regions were again smaller in females than in males, but in only non-bruxist individuals. These gender-related differences in FA values may be due to differences in the muscle forces or metabolic activities and hormones between females and males, each of which may have an affect bones. In case of difference, it is remarkable that females have smaller FA values than males. However, since no difference was found in the FA values obtained from the condyles, angulus, and corpus of girls and boys in the study of Kolcakoglu et al. [32], which consisted of children aged 5–11 years, it can be thought that this difference occurs with adulthood. It is known that chewing forces are higher in males, and the literature has reported higher FA values in males, consistent with this study. Although these results, the assumption that an increase in occlusal loads causes an increase in FA values will not be correct as some results show the opposite. Yasar and Akgunlu [37], compared the FAs of dentate and edentulous bone in the premolar-molar region of the mandible and reported that dentate regions had significantly lower FA values than edentulous regions.

How the FD of bone is formed seems quite complex and it is obviously multifactorial. We found no significant difference in the FA values obtained from the condyle, corpus, and angulus regions of bruxist patients compared to the FA values in the same regions of non-bruxist patients. As we mentioned above, there are studies in the literature reporting significant differences between bruxist and non-bruxist groups in some of these regions. There is no consensus on the direction of the difference in these studies. Some researchers reported lower FA values in bruxist patients, while others reported higher values compared to the control groups. In addition, in almost all of these studies, the difference between bruxist and non-bruxist groups was limited to a few regions, and no general difference was found between the bruxist and control groups in the regions included in these studies. The authors of this study report that bruxism did not cause a significant change in the FDs of the bone in the mandibular condyle, corpus, and angulus region in the age group examined, and that gender had a significant effect on the FDs of these regions.

In this study, a significant correlation was determined between age and FA of the mandibular corpus only. FA values of the mandibular corpus increased with increasing age. The youngest participant was 18, and oldest was 43 years old, and the mean age of the sample was 27.55 (± 7.022) years. The sample consisted of young adults, and they were below the age considered at risk for osteoporosis. Due to this limited age range in this study to avoid the effects of age-related changes in bone, it was not surprising that there was no remarkable change in the measurements with age. Similarly, in another study whose sample was limited to the age range of 21–40 years, a weak negative correlation was found between age and only right condyle FA measurements, while no correlation was found with age in the left condyle and other regions [23]. In another study [28] in which the age range was between 18 and 45, the correlation between age and FA values was nonsignificant. However, in non-bruxist children, a negative correlation was reported between age and FA values of the condyle, that is, FA values decrease as the age increases from 5 to 11 [32].

In the literature, limited studies examined MCI, MI, and PMI indices in bruxist patients, to discuss our results. And one study was excluded due to inconsistency in terminology [38]. For MCI, Isman et al. [30] and Yılmaz et al. [39] reported more common healthy cortex (MCI-1 category) in non-bruxist individuals and more endosteal cortical residues and porosity in bruxist individuals. Isman et al. [30] although they found high MI in bruxist individuals, thought that increased masticatory force initiating resorption of the cortical bone. However, in the present study like study of, Eninanç et al. [40] no relationship was found between MCI and bruxism. In this present study, MCI was independent of age like previous studies [30, 40] and significantly lower in females (*p* = .000). This means that in our sample, healthy cortex was more common in females than males. This result is consistent with the study that reported type C1 cortex was more common in females in both bruxist and non-bruxist groups [40]. The age distribution of the participants in this study is very similar to that in our study, with a range of 18–45 and mean of 26.34 ± 6.92. Thus, the osteoporotic effect of menopause, which might affect the outcome, was excluded in these study groups.

In this study, cortical thinning was detected in the mental region of bruxist patients, but the difference with non-bruxist patients did not exceed statistical significance (*P* = .083). Previous two studies [30, 40] reported significantly higher MI in bruxists, unlike our result. While Isman [30], reported a significant correlation between the MI and age, present study found no correlation, as in the study of Eninanç et al. [40]. The association of MI with gender was not significant, consistent with study of Isman [30], but Eninaç et al. [40], was reported significantly lower MI values for females.

Similar to previous studies, no significant relationship was found between bruxism and PMI [30, 40]. PMI was significantly lower in males (*p* = .004). We attribute this to the absence of a significant difference in cortical thickness in the mental region, and to the higher vertical dimension of the mandible in men. However, in some previous studies, no relationship was reported between PMI and gender [30, 40]. PMI didn’t correlate in the present study with age, consistent with previous studies [30, 40].

While detectable differences in bones that will affect indices may take a long time, changes in bone mineralization can be expected as a result of shorter-term factors. In the study, the appropriate sample size was created under the guidance of previous studies and power analysis, and the study and control groups were meticulously formed on a scientific basis. The limitation of the study is that the type (sleep, awake or mixed) and duration of bruxism in patients was not taken into account.

There may be a significant difference between the control group and patients with long-term bruxism. However, no significant difference was also found between bruxist and non-bruxist patients in FA values, which is thought to be a precursor to visible changes in bone. However, examining the groups separated according to the duration of bruxism in future studies so that statistical tests can reveal clear results will enable us to be sure of the effect of bruxism on the mandible.

## Conclusions

This study reports that bruxism doesn’t cause a significant change in the FDs of the bone in the mandibular condyle, corpus and angulus region, and doesn’t significantly change MCI, MI, and PMI. While bruxist individuals did not differ significantly from the control group according to the indices, FA did not reveal a different result. However, FA of the condyle and angulus of the mandible, as well as MCI and PMI are significantly affected by gender and PR, which was preferred for this study because of its advantages over 3D imaging, allows for this determination. In females, FA values were significantly lower in the condyle and angulus regions, PMI was significantly higher, and the prevalence of healthy cortex was significantly more frequent. In the light of these results, it was considered that MCI, MI, PMI, and FA values could not be predictive in the diagnosis of bruxism. Further studies taking into account the type and duration of bruxism may contribute to explaining the changes in trabecular bone.

## Data Availability

All the datasets used and analyzed during the current study are available from the corresponding author on reasonable request.

## Abbreviations

PR: Panoramic radiography
FA: Fractal analysis
FD: Fractal dimension
MCI: Mandibular cortical index
MI: Mental index
PMI: Panoramic mandibular index

## References

[1] Lobbezoo F, Ahlberg J, Glaros AG, Kato T, Koyano K, Lavigne GJ (2013). Bruxism defined and graded: an international consensus. J Oral Rehabil..

[2] Melo G, Duarte J, Pauletto P, Porporatti AL, Stuginski-Barbosa J, Winocur E (2019). Bruxism: an umbrella review of systematic reviews. J Oral Rehabil.

[3] Jiménez-Silva A, Peña-Durán C, Tobar-Reyes J, Frugone-Zambra R (2017). Sleep and awake bruxism in adults and its relationship with temporomandibular disorders: a systematic review from 2003 to 2014. Acta Odontol Scand.

[4] Manfredini D, Lobbezoo F (2021). Sleep bruxism and temporomandibular disorders: a scoping review of the literature. J Dent.

[5] Glaros AG, Rao SM, Bruxism (1977). A critical review. Psychol Bull.

[6] Gustavsson A, Thorsen K, Nordström P (2003). A 3-year longitudinal study of the effect of physical activity on the accrual of bone mineral density in healthy adolescent males. Calcif Tissue Int.

[7] Daly RM, Dalla Via J, Duckham RL, Fraser SF, Helge EW. Exercise for the prevention of osteoporosis in postmenopausal women: an evidence-based guide to the optimal prescription. Braz J Phys Ther 2019 Mar-Apr 23(2):170–80. doi: 10.1016/j.bjpt.2018.11.011. 10.1016/j.bjpt.2018.11.011 PMC6429007 30503353

[8] Inoue M, Ono T, Kameo Y, Sasaki F, Ono T, Adacheti T (2019). Forceful mastication activates osteocytes and builds a stout jawbone. Sci Rep.

[9] Walters M, Crew M, Fyfe G (2019). Bone surface Micro-Topography at Craniofacial Entheses: insights on osteogenic adaptation at muscle insertions. Anat Rec (Hoboken).

[10] European Commission (2004). European guidelines on Radiation Protection in Dental Radiology.

[11] Kinalski MA, Boscato N, Damian MF (2020). The accuracy of panoramic radiography as a screening of bone mineral density in women: a systematic review. Dentomaxillofac Radiol.

[12] Munhoz L, Morita L, Nagai AY, Moreira J, Arita ES (2021). Mandibular cortical index in the screening of postmenopausal at low mineral density risk: a systematic review. Dentomaxillofac Radiol.

[13] Larheim TA, Svanaes DB (1986). Reproducibility of rotational panoramic radiography: mandibular linear dimensions and angles. Am J Orthod Dentofacial Orthop.

[14] Langland OE, Langlais RP, Preece JW (2002). Principles of Dental Imaging.

[15] Lopes R, Betrouni N (2009). Fractal and multifractal analysis: a review. Med Image Anal.

[16] Kato CN, Barra SG, Tavares NP, Amaral TM, Brasileiro CB, Mesquita RA (2020). Use of fractal analysis in dental images: a systematic review. Dentomaxillofac Radiol.

[17] Cavalcante DS, Silva PGB, Carvalho FSR, Quidute ARP, Kurita LM, Cid AMPL (2022). Is jaw fractal dimension a reliable biomarker for osteoporosis screening? A systematic review and meta-analysis of diagnostic test accuracy studies. Dentomaxillofac Radiol.

[18] Klemetti E, Kolmakov S, Kroger H (1994). Pantomography in assessment of the osteoporosis risk group. Scand J Dent Res.

[19] Ledgerton D, Horner K, Devlin H, Worthington H (1997). Panoramic mandibular index as a radiomorphometric tool: an assessment of precision. Dentomaxillofac Radiol.

[20] Benson BW, Prihoda TJ, Glass BJ (1991). Variations in adult cortical bone mass as measured by a panoramic mandibular index. Oral Surg Oral Med Oral Pathol.

[21] White SC, Rudolph DJ (1999). Alterations of the trabecular pattern of the jaws in patients with osteoporosis. Oral Surg Oral Med Oral Pathol Oral Radiol Endod.

[22] Landis JR, Koch GG (1977). The measurement of observer agreement for categorical data. Biometrics.

[23] Gulec M, Tassoker M, Ozcan S, Orhan K (2021). Evaluation of the mandibular trabecular bone in patients with bruxism using fractal analysis. Oral Radiol.

[24] Lee HJ, Kim SJ, Lee KJ, Yu HS, Baik HS (2017). Repeated injections of botulinum toxin into the masseter muscle induce bony changes in human adults: a longitudinal study. Korean J Orthod.

[25] Qin YX, Lam H, Ferreri S, Rubin C (2010). Dynamic skeletal muscle stimulation and its potential in bone adaptation. J Musculoskelet Neuronal Interact.

[26] Cianferotti L, Brandi ML (2014). Muscle-bone interactions: basic and clinical aspects. Endocrine.

[27] Southard TE, Southard KA, Jakobsen JR, Hillis SL, Najim CA (1996). Fractal dimension in radiographic analysis of alveolar process bone. Oral Surg Oral Med Oral Pathol Oral Radiol Endod.

[28] Eninanç İ, Yalçın Yeler D, Çınar Z (2021). Investigation of mandibular fractal dimension on digital panoramic radiographs in bruxist individuals. Oral Surg Oral Med Oral Pathol Oral Radiol.

[29] Türp JC, Simonek M, Dagassan D (2021). Bone apposition at the mandibular angles as a radiological sign of bruxism: a retrospective study. BMC Oral Health.

[30] Isman O (2021). Evaluation of jaw bone density and morphology in bruxers using panoramic radiography. J Dent Sci.

[31] Casazza E, Ballester B, Philip-Alliez C, Raskin A. Evaluation of mandibular bone density in bruxers: the value of panoramic radiographs. Oral Radiol. 2022 Apr;19. 10.1007/s11282-022-00612-3. 10.1007/s11282-022-00612-3 35438407

[32] Kolcakoglu K, Amuk M, Sirin Sarıbal G (2022). Evaluation of mandibular trabecular bone by fractal analysis on panoramic radiograph in paediatric patients with sleep bruxism. Int J Paediatr Dent.

[33] Kurt MH, Yilmaz S, Evli C, Karahan S (2023). Comparative evaluation of trabecular bone structures of Bruxist and Non-Bruxist individuals with bone apposition in the Mandible Angle Region by Fractal Analysis. J Oral Rehabil.

[34] Arsan B, Köse TE, Çene E, Özcan İ (2017). Assessment of the trabecular structure of mandibular condyles in patients with temporomandibular disorders using fractal analysis. Oral Surg Oral Med Oral Pathol Oral Radiol.

[35] Dias GM, Bonato LL, Guimarães JP, Silva JN, Ferreira LA, Grossmann E et al. A Study of the Association Between Sleep Bruxism, Low Quality of Sleep, and Degenerative Changes of the Temporomandibular Joint. J Craniofac Surg. 2015; 26(8):2347-50. doi: 10.1097/SCS.0000000000002084. PMID: 26501968. 10.1097/SCS.0000000000002084 26501968

[36] Raphael KG, Tadinada A, Bradshaw JM, Janal MN, Sirois DA, Chan KC (2014). Osteopenic consequences of botulinum toxin injections in the masticatory muscles: a pilot study. J Oral Rehabil.

[37] Yasar F, Akgünlü F. Fractal dimension and lacunarity analysis of dental radiographs. Dentomaxillofac Radiol. 2005;34(5):261-7. doi: 10.1259/dmfr/85149245. PMID: 16120874. 10.1259/dmfr/85149245 16120874

[38] Al-Dwairi ZN, Al-Daqaq ANF, Kielbassa AM, Lynch E (2017). Association between oral tori, occlusal force, and mandibular cortical index. Quintessence Int.

[39] Yilmaz S, Kurt MH, Durmaz Yilmaz OM, Karahan S, Canger EM. A new perspective for radiologic findings of bruxism on dental panoramic radiography. Oral Radiol. 2022 Dec 12. doi: 10.1007/s11282-022-00667-2Epub ahead of print. PMID: 36504381. 10.1007/s11282-022-00667-2 36504381

[40] Eninanc I, Yeler DY, Cinar Z. Evaluation of the effect of bruxism on mandibular cortical bone using radiomorphometric indices on panoramic radiographs. Niger J Clin Pract. 2021 Nov; 24(11):1742-1748. 10.4103/njcp.njcp_71_21. 10.4103/njcp.njcp_71_21 34782517

